# A branched‐chain amino acid‐based metabolic score can predict liver fat in children and adolescents with severe obesity

**DOI:** 10.1111/ijpo.12739

**Published:** 2020-10-14

**Authors:** Julia Lischka, Andrea Schanzer, Azadeh Hojreh, Ahmed Ba Ssalamah, Chike Bellarmine Item, Charlotte de Gier, Nina‐Katharina Walleczek, Thomas F. Metz, Ivana Jakober, Susanne Greber‐Platzer, Maximilian Zeyda

**Affiliations:** ^1^ Clinical Division of Pediatric Pulmonology, Allergology and Endocrinology, Department of Pediatrics and Adolescent Medicine Medical University of Vienna Vienna Austria; ^2^ Comprehensive Center for Pediatrics, Medical University of Vienna Vienna Austria; ^3^ Department of Biomedical Imaging and Image‐guided Therapy Medical University of Vienna Vienna Austria

**Keywords:** biomarker, branched‐chain amino acids, pediatric obesity, metabolic score, non‐alcoholic fatty liver disease

## Abstract

**Background:**

Eighty percent of adolescents with severe obesity suffer from non‐alcoholic fatty liver disease (NAFLD). Non‐invasive prediction models have been tested in adults, however, they performed poorly in paediatric populations.

**Objective:**

This study aimed to investigate novel biomarkers for NAFLD and to develop a score that predicts liver fat in youth with severe obesity.

**Methods:**

From a population with a BMI >97th percentile aged 9‐19 years (*n* = 68), clinically thoroughly characterized including MRI‐derived proton density fat fraction (MRI‐PDFF), amino acids and acylcarnitines were measured by HPLC‐MS.

**Results:**

In children with NAFLD, higher levels of plasma branched‐chain amino acids (BCAA) were determined. BCAAs correlated with MRI‐PDFF (*R* = 0.46, *p* < .01). We identified a linear regression model adjusted for age, sex and pubertal stage consisting of BCAAs, ALT, GGT, ferritin and insulin that predicted MRI‐PDFF (*R* = 0.75, *p* < .01). ROC analysis of this model revealed AUCs of 0.85, 0.85 and 0.92 for the detection of any, moderate and severe steatosis, respectively, thus markedly outperforming previously published scores.

**Conclusion:**

BCAAs could be an important link between obesity and other metabolic pathways. A BCAA‐based metabolic score can predict steatosis grade in high‐risk children and adolescents and may provide a feasible alternative to sophisticated methods like MRI or biopsy in the future.

## 1 INTRODUCTION

Despite major public health efforts to avoid overnutrition, the global prevalence of obesity is still rising, and concomitantly, the prevalence of non‐alcoholic fatty liver disease (NAFLD).^1^ NAFLD is an important predictor for mortality^2^ and is known to increase the risk of type 2 diabetes, cardiovascular disease and dyslipidemia,^3^, ^4^ already in youth with obesity.^3^, ^5^ NAFLD is often the first comorbidity that arises in obesity^6^ and the degree of liver fat content relates to the grade of metabolic disease,^7^, ^8^, ^9^ underlining the importance of steatosis as an early indication of metabolic disease. However, the underlying pathomechanisms are poorly understood.^9^

The need for characterization of a high‐risk group for NAFLD and targeted approaches early in life was formally acknowledged by the Committee on prevention of obesity in children and youth of the U.S. Institute of Medicine.^10^ Reliable quantitative determination of liver fat content, however, is based on either biopsy or sophisticated imaging techniques^11^ only available in specialized centres. Thus, there is a vital need for reliable but simple biomarkers for quantification of liver fat content. To this objective, biochemical markers and routine measures have been evaluated. GGT and ALT, known measures of liver disease, have been proposed as independent predictors of NAFLD.^12^ However, Wong et al. found that ALT levels show a high variability on repeated testing and do not reliably diagnose NAFLD nor correlate with histologic grading.^13^ Therefore, metabolic risk factors were proposed as the basis of NAFLD evaluation, but most studies are limited by evaluating NAFLD solely as a dichotomous parameter.^14^, ^15^, ^16^, ^17^ Liver fat content seems to directly relate to the degree of metabolic disease and should therefore be quantitatively determined.^18^, ^19^

Metabolomics approaches showed that amino acid patterns are more strongly associated with metabolic health than traditional laboratory and also lipid markers.^20^ Big cohorts identified circulating branched chain amino acids (BCAAs) to be chronically elevated in individuals with obesity. Compelling evidence derived from rodent studies highlights their causal connection to the risk of T2D and insulin resistance and cardiovascular disease.^21^, ^22^, ^23^ Hence, elevated BCAAs are characteristic for deteriorated metabolic health^20^, ^22^, ^24^, ^25^ already early in life^16^, ^18^ and predict future disease risk.^16^, ^22^, ^26^ BCAAs have been shown to promote intrahepatic fat accumulation in an animal model^27^ and are elevated in human individuals with NAFLD,^14^, ^15^, ^16^, ^17^, ^18^, ^19^ suggesting a link between impaired amino acid metabolism and liver fat accumulation.^17^ The aetiology and pathophysiological pathways of increased BCAA levels in obesity is still unclear, but may involve chronic low grade inflammation by inducing pro‐inflammatory gene expression in adipose tissue,^28^, ^29^ thereby further deteriorating obesity effects on metabolic health, also in cardiovascular^24^, ^25^ and liver disease.^27^

Substantial evidence that links BCAA dysmetabolism to a metabolically unhealthy phenotype with obesity including steatosis, hepatic injury or inflammation^14^, ^15^, ^16^, ^17^, ^18^, ^19^, ^27^, ^30^ has been published. Therefore, defining a BCAA‐related metabolic signature that indicates the liver fat content could not only help to identify and monitor patients with NAFLD and increased cardiometabolic risk,^5^, ^21^, ^23^, ^24^, ^25^, ^26^ but also to elucidate the underlying pathomechanisms.

Early interference in paediatric patients to prevent progression of NAFLD and other obesity‐related disorders is highly desirable.^2^, ^10^ Furthermore, the pathogenesis of NAFLD in children and adolescents is much less investigated and may significantly differ compared to adults.^31^ Therefore, studies in paediatric patients are strongly needed. The only existing study with an accurate quantification of liver fat content by MRI analyzing amino acid levels in children and adolescents investigated a majority of non‐Caucasian individuals.^18^ Plasma concentrations of BCAAs were shown to be associated with intra‐hepatic fat content independently of the degree of obesity and insulin resistance.^18^ Although these results may not directly be applicable to European cohorts,^9^, ^32^ they provide a strong indication for BCAAs to be investigated in Caucasian paediatric patients in relation to NAFLD. Same accounts for acylcarnitines, which are not only linked to fatty acid metabolism but some of the shorter forms also to BCAA metabolism. Interestingly, the latter have been shown to be linked to insulin resistance.^15^, ^21^, ^26^

To fill these gaps in research in the high‐risk group of youths with severe obesity and to further contribute to elucidation of the complex mechanisms leading to paediatric NAFLD, we investigated whether circulating amino acid levels are associated with liver fat content as measured by MRI in children and adolescents with severe obesity. Moreover, a plethora of amino acids, acylcarnitines and established clinical as well as experimental markers for liver function, metabolic state, and inflammation was considered for development of a simple and thus practicable score to predict liver fat content. The resulting score includes BCAAs, ALT, GGT, ferritin and insulin to predict liver fat content in paediatric patients with severe obesity with high accuracy.

## 2 METHODS

### 2.1 Patients

Patients attending the outpatient clinic for obesity and lipid‐metabolic disorders at the Department of Pediatrics and Adolescent Medicine at the Medical University of Vienna with a BMI above the 97th percentile (referred to as “severe obesity”^33^, ^34^ throughout this manuscript) were prospectively enrolled in this study. Eligible for this study were all patients between 9 to 19 years old. Patients were excluded if one or more of the following exclusion criteria were met: Chromosomal aberrations and syndromes associated with obesity (eg, Prader‐Willi‐Syndrome), treatment with drugs associated with elevated liver enzymes and if other causes for liver disease were present (eg, Wilson's disease, hepatitis infection). Of 94 eligible patients, 68 were included in the study. Twenty‐six patients were excluded, because of incompliance with study protocol. Subsequently, a second cohort of 32 paediatric patients was recruited for independent validation.

All study participants underwent physical examination including Tanner stage. Medical history, clinical and laboratory data was collected for all study participants. Anthropometric measures were taken by standardized methods by the same two nurses throughout the study. Body mass index (BMI, kg/m^2^) and the respective percentiles and SD scores were calculated according to Kromeyer‐Hauschild et al.^35^ Body fat in percent (body fat%) was determined by bioelectrical impedance analysis (BIA). Serum and plasma samples were taken in an overnight fasting state and, for non‐routine parameters, frozen at −80°C until analysis. Homeostasis model of insulin resistance (HOMA‐IR) was calculated according to Matthews et al.: fasting glucose (mmol/L) * fasting insulin (mU/L)/22.5.^36^

### 2.2 Liver fat content

Accumulation of liver fat was quantified by magnetic resonance imaging‐proton density fat fraction (MRI‐PDFF). MRI scan was performed at the Department of Biomedical Imaging and Image‐guided Therapy, Medical University of Vienna, on a 1.5 Tesla MR‐Scanner Siemens Magnetom Aera. Image data was evaluated using a PACS (picture archiving and communication system, IMPAX EE, Agfa Healthcare, Mortsel, Belgium) on a diagnostic grey‐scale monitor (Barco MDCG‐3120, Brussels, Belgium). For MRI‐PDFF calculation, one MR‐slice of in‐phase sequence and the corresponding slice of opposed‐phase sequence were used. The quality of MR‐images was evaluated by a senior radiologist (A.H.). In the case of severe motion artefacts, which reduce the diagnostic image quality, patients would have been excluded, which was never the case. MRI‐PDFF was calculated according to Sirlin et al.^37^ and was calculated and interpreted by the same senior radiologist (A.H.) in all patients. Steatosis was defined as a MRI‐PDFF of 5.1% or more. Mild steatosis (grade 1) was specified as a MRI‐PDFF below 14.1%, moderate steatosis (grade 2) below 28.0% and a MRI‐PDFF of 28.0% and above was defined as severe steatosis (grade 3).^38^, ^39^

### 2.3 Amino acids and acylcarnitines

Plasma amino acid and acylcarnitine concentrations were determined on a Waters Acquity UPLC‐coupled Xevo TQD mass spectrometer using non‐derivatized a semi‐quantitative kit for dried blood spots from Chromsystems (Gräfeling, Germany), which was modified by directly pipetting 1.3 μL plasma, sampled and stored as described above, into extraction buffer. For confirmation, amino acids were additionally quantified from fresh plasma of 30 of the patients using the EZ:faast kit from Phenomenex (Torrance, CA) on a Waters Q‐Micro HPLC‐coupled mass spectrometer with essentially same results (not shown). BCAA concentrations were calculated by addition of valine, leucine and isoleucine values.

### 2.4 Calculation of known scores

Enhanced liver fibrosis (ELF) test,^40^ fatty liver index (FLI),^41^ GSG index,^14^ hepatic steatosis index (HSI),^42^ visceral adiposity index (VAI)^43^ and triglycerides glucose (TyG) index^44^ were calculated exactly as described in the given references.

### 2.5 Statistics

In order to perform group‐wise comparisons, steatosis was categorized as described above. Ordinal scaled data was analyzed with the ANOVA. For normally distributed variables, correlation among parameters was assessed by Pearson correlation analyses. For skewed variables, Spearman correlation was calculated.

Analyzed parameters included: BMI *z*‐score, body fat, waist circumference, hip circumference, ferritin, uric acid, platelets, alkaline phosphatase, GGT, ALT, AST, bilirubin, triglycerides, total cholesterol, fasting glucose, insulin, HOMA‐IR, CRP, IL6, procalcitonin, TNFα, CK‐18, BCAAs, the amino acids alanine, aspartic acid, glutamic acid, glycine, proline, tyrosine, arginine, phenylalanine, ornithine, citrulline and acylcarnitines propionylcarnitine (C3), butyrylcarnitine (C4) and isovalerylcarnitine (C5), hexanoylcarnitine (C6), octanoylcarnitine (C8), decanoylcarnitine (C10), dodecanoylcarnitine (C12), tetradecanoylcarnitine (C14), hexadecanoylcarnitine (C16) and stearoylcarnitine (C18). Partial least squares regression was performed to identify the statistically most important variables: Variables with a variable importance in projection (VIP) >1 were selected for further analysis.^45^, ^46^ In the next step, variables were entered into a multiple linear regression analysis to assess independent association. In the linear regression model, each indicator was assessed as an independent variable and MRI‐PDFF as dependent variable. Covariates were selected from known predictors of childhood obesity.

The predictive value of our model was assessed by the area under the receiver operating characteristic curve (AUROC, c statistic). The score characteristics, sensitivity and specificity, were calculated. In order to estimate the clinical use of our model, positive predictive value (PPV) and negative predictive value (NPV) were calculated to determine the probability of the disease in the individual patient.

All analyses were conducted without stratification because we found no interaction attributable to sex or age. A two‐sided *p*‐value under .05 was considered statistically significant. All statistical analyses were performed using IBM SPSS Statistics for Windows, version 25 (IBM Corp., Armonk, New York).

### 2.6 Ethics

The study protocol was approved by the ethics committee of the Medical University of Vienna (No. 1638/2019) and conducted according to the Helsinki declaration guidelines. Written informed consent was obtained from all participants as well as their legal guardians prior to all study procedures.

## 3 RESULTS

Characteristics of the study population are shown in Table 1. Sixty‐eight patients with mean age of about 13 years completed MRI evaluation of the liver and were included in the study. All were of Caucasian ethnicity. Of the investigated parameters, HOMA‐indices, liver transaminases, GGT, total cholesterol, insulin, triglycerides, ferritin, PCT, TNFα and CK‐18 significantly correlated with liver fat content (*p*‐value ≤ .02 for all, Table S1).

**TABLE 1:** Anthropometric and clinical characteristics of study subjects

		No steatosis (*n* = 33)	Mild steatosis (*n* = 16)	Moderate steatosis (*n* = 18)	Severe steatosis (*n* = 12)	*p*‐value
Gender	(female/male)	14/19	6/10	4/14	3/9	n.s.
Age		13.00 (3.00)	13.00 (3.00)	13.00 (3.00)	13.00 (2.00)	n.s.
BMI *z*‐score		5.89 (9.95)	4.79 (8.42)	9.71 (13.87)	7.77 (11.13)	n.s.
Waist circumference (cm)		109.56 (23.05)	107.14 (19.23)	119.09 (29.57)	120.44 (27.78)	n.s.
Hip circumference (cm)		110.97 (14.95)	107.27 (13.17)	109.51 (11.99)	114.35 (20.45)	n.s.
BIA (body fat%)		40.29 (7.20)	41.66 (6.32)	38.88 (5.24)	40.14 (6.86)	n.s.
HOMA‐IR		2.96 (1.95, 4.43)	5.26 (3.06, 8.44)	5.34 (5.00, 6.49)	6.84 (5.41, 9.82)	<.01
HOMA‐β		3.72 (2.37, 5.38)	7.14 (3.85, 11.10)	6.87 (5.84, 9.61)	8.12 (6.80, 9.99)	<.01
Fasting glucose (mg/dl)		81.00 (9.43)	82.38 (11.28)	87.09 (12.07)	83.35 (7.60)	n.s.
Insulin (μU/ml)		14.16 (8.77)	29.98 (20.83)	25.86 (22.77)	26.76 (17.8)	.02
Ferritin (μg/l)		44.11 (21.66)	57.38 (36,24)	60.78 (30,82)	144.93 (149.71)	.01
Platelet count (10̂9/L)		277.47 (54.00)	303.81 (66.00)	315.06 (78.00)	280.33 (49.40)	n.s.
AP (U/l)		162.45 (85.47)	177.31 (61.54)	173.94 (80.83)	188.25 (82.17)	n.s.
GGT (U/l)		19.27 (11.37)	56.67 (125.98)	30.28 (25.66)	42.58 (28.74)	<.01
ALT (U/l)		29.36 (9.89)	46.75 (42.83)	52.39 (32.27)	116.08 (97.31)	<.01
AST (U/l)		26.33 (5.73)	36.44 (17.65)	36.33 (15.38)	61.00 (37.38)	<.01
Triglycerides (mg/dl)		89.00 (71.00, 122.00)	131.00 (104.00, 184.00)	115.00 (89.00, 221.00)	145.00 (98.00, 212.00)	<.01
Total cholesterol (mg/dl)		155.00 (141.00, 187.00)	164.00 (160.00, 188.00)	163.00 (153.00, 202.00)	181.00 (158.00, 218.00)	n.s.
HDL‐C (mg/dl)		45.00 (38.00, 48.00)	42.00 (31.00, 50.00)	43.00 (39.00, 46.00)	39.00 (33.00, 43.00)	n.s.
Uric acid (mg/dl)		5.25 (1.77)	5.99 (1.78)	4.31 (2.69)	5.52 (2.77)	n.s.
ELF test		8.60 (0.79)	8.63 (0.58)	8.90 (0.55)	8.61 (0.72)	n.s.
CRP (mg/dl)		0.72 (0.28, 1.24)	0.70 (0.47, 0.97)	0.33 (0.18, 1.27)	0.85 (0.25, 3.33)	n.s.
IL‐6 (pg/ml)		3.32 (1.50, 5.01)	2.88 (1.50, 4.58)	2.90 (1.50, 3.64)	2.90 (2.21, 4.25)	n.s.
Procalcitonin (ng/ml)		0.04 (0.03, 0.06)	0.05 (0.03, 0.05)	0.05 (0.04, 0.09)	0.07 (0.04, 0.09)	n.s.
TNFα (pg/ml)		1.05 (0.90, 1.25)	1.25 (0.95, 1.60)	1.00 (0.75, 1.50)	1.55 (1.30, 1.65)	.03
CK‐18 (U/l)		92.48 (83.57, 134.53)	101.10 (83.57, 204.63)	107.87 (99.80, 148.40)	212.33 (141.73, 385.79)	<.01
BCAAs (μmol/l)		461.62 (81.14)	490.29 (108.74)	491.40 (60.29)	545.56 (80.15)	<.01
C3 (μmol/l)		0.48 (0.16)	0.60 (0.28)	0.72 (0.28)	0.68 (0.20)	.01
C4 (μmol/l)		0.20 (0.08)	0.24 (0.12)	0.28 (0.12)	0.36 (0.12)	<.01
C5 (μmol/l)		0.12 (0.04)	0.16 (0.04)	0.20 (0.16)	0.20 (0.32)	n.s.

*Note:* Values are means and (SD) for normally distributed variables and median (25th, 75th percentile) for skewed variables. *p*‐values less than .05 was considered significant and were determined by ANOVA or Kruskal Wallis test, respectively.

Abbreviations: AP, alkaline phosphatase; BMI, body mass index; BIA (body fat%), body fat in % determined by bioelectrical impedance analysis; ELF, enhanced liver fibrosis; HOMA‐IR, homeostatic model assessment of insulin resistance; HOMA‐β, homeostatic model assessment of liver insulin resistance.

**FIGURE 1:**
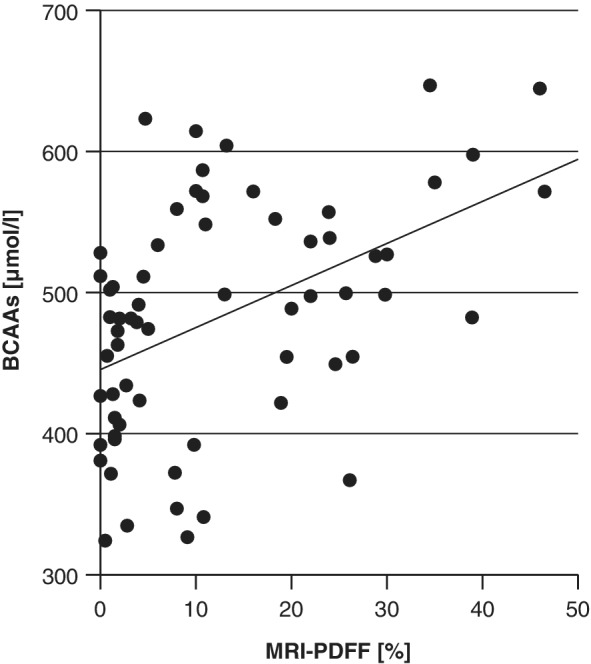
Correlation of BCAAs with MRI‐PDFF. BCAAs significantly correlate with liver fat (*p*‐value < .01, *R* = 0.46)

To assess the metabolic profile of the patients, 10 amino acids and 10 acylcarnitines were determined and exploratively analyzed for associations. In particular BCAAs and related acylcarnitine concentrations appeared to be linked to fatty liver: The correlation of BCAAs with liver fat content (*p*‐value < .01, *R* = 0.46) also after adjustment for gender, age and pubertal stage is shown in Figure 1. Additionally, the ANOVA showed significant differences in BCAA levels between steatosis stages (*p*‐value .03). Also concentrations of acylcarnitines C3 and C4 significantly differed between the groups (Table 1), moreover, acylcarnitines C3, C4 and C5, byproducts of BCAA degradation, correlated with MRI‐PDFF (Table 2), essentially confirming the BCAA results. Moreover, BCAAs and C3, C4, C5 significantly correlated with ALT and CK‐18 as markers for advanced steatosis (Table 2). Also HOMA‐β and HOMA‐IR correlated with BCAAs, C3, C4 and C5 (Table 2).

**TABLE 2:** Correlation between BCAAs, acylcarnitines and markers of NAFLD. BCAAs and their degradation products C3, C4 and C5 correlate with hepatic fat content, liver enzymes, inflammatory marker TNFα and hepatocyte apoptosis marker CK‐18

	MRI‐PDFF		ALT (U/l)		TNFα		CK‐18		HOMA‐IR	
	*R*	Sig.	*R*	Sig.	*R*	Sig.	*R*	Sig.	*R*	Sig.
C3	0.43	<0.01	0.40	<0.01	0.24	0.09	0.26	0.04	0.31	0.02
C4	0.42	<0.01	0.42	<0.01	0.59	<0.01	0.26	0.04	0.29	0.03
C5	0.25	0.04	0.40	<0.01	0.23	0.11	0.45	<0.01	0.30	0.02
BCAAs	0.46	<0.01	0.46	<0.01	0.20	0.20	0.30	0.02	0.49	<0.01

### 3.1 Linear regression model

**FIGURE 2:**
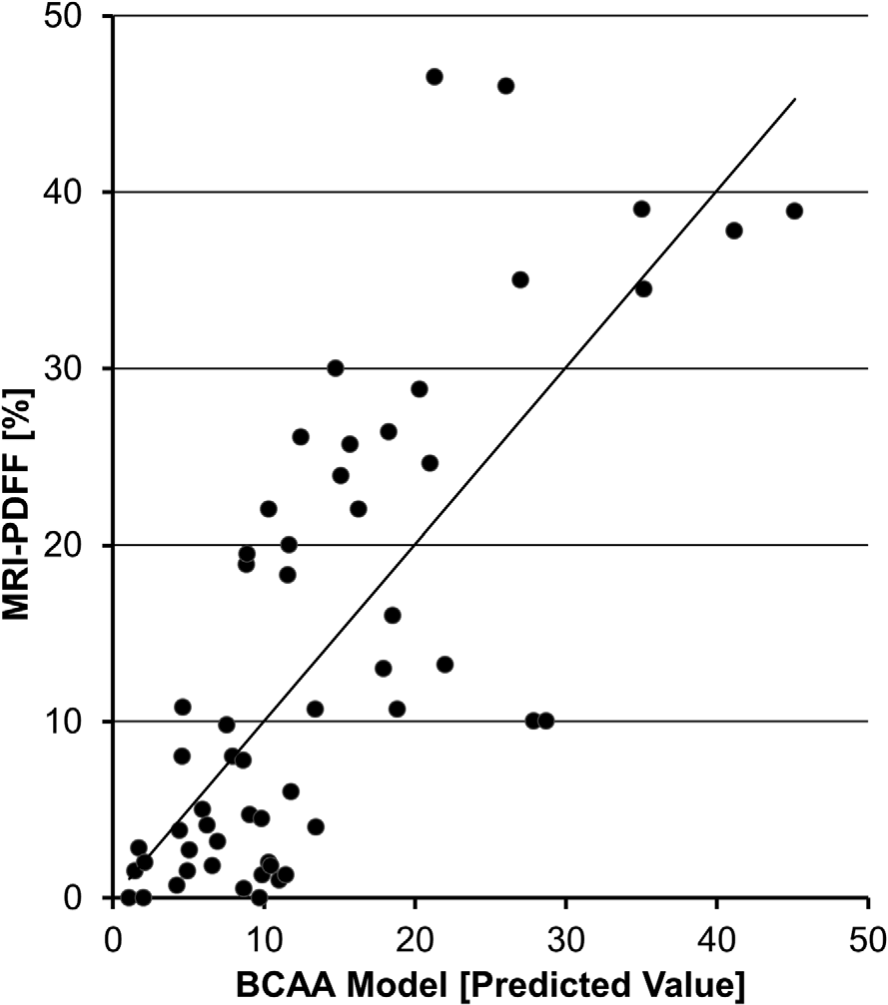
Correlation of MRI‐PDFF and predicted hepatic fat content. The BCAA‐based metabolic score predicts liver fat content in youth with severe obesity. The final model to predict hepatic fat content (MRI‐PDFF) consisted of the parameters BCAAs, ALT, GGT and insulin (*p*‐value < .01; *R* = 0.75, *R* ^2^ = 0.57)

Despite the strong and significant correlation of BCAAs and some acylcarnitines with liver fat content, their analytical value is limited when considered in isolation. Therefore, a linear regression model was built as detailed in the Material and Methods section. Parameters primarily taken into account included anthropometric measures, routine laboratory parameters (eg, liver enzymes), markers of inflammation (eg, TNFα, IL‐6, CRP) and markers of liver status (eg, uric acid, procalcitonin, ferritin) as well as amino acids and acylcarnitines. The model was adjusted for age, sex and pubertal stage. The final model to predict hepatic fat content (MRI‐PDFF) consisted of the parameters BCAAs, ALT, GGT and insulin and is defined as follows (*p*‐value < .01; *R* = 0.75, *R* ^2^ = 0.57, Figure 2): Predicted MRI‐PDFF = BCAAs (μmol/l) * 0.03 + ALT (U/l) * 0.144 + GGT (U/l) * (−0.208) + Ferritin (μg/l) * 0.041 + Insulin (μU/ml) * 0.313‐14.04.

**FIGURE 3:**
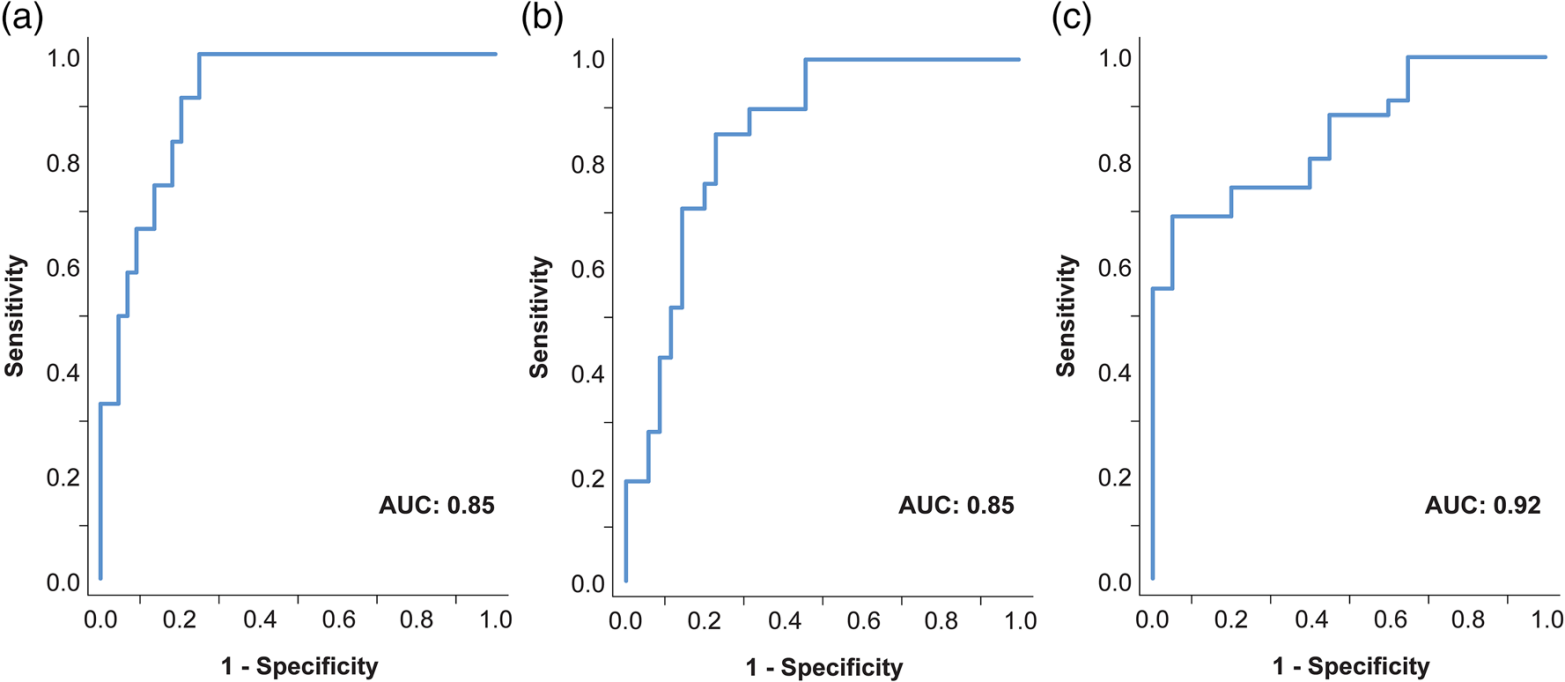
ROC curves evaluating the performance of the BCAA‐based metabolic score. ROC curves were calculated to determine accuracy of detecting (A) mild steatosis, (B) moderate steatosis and (C) severe steatosis, respectively. The BCAA‐based metabolic score showed an excellent diagnostic accuracy with an AUC > 0.8 for all steatosis grades. Diagnostic accuracy was highest for severe steatosis (AUC 0.92). *n* = 59 (68 subjects were analyzed, 9 were excluded due to missing data)

Accuracy of the model was assessed by the c‐statistic (AUROC). ROC curves are shown in Figure 3. AUC was 0.85 (95%CI 0.76‐0.95) for the diagnosis of any steatosis (MRI‐PDFF >5.1%) and 0.85 (95%CI 0.76‐0.95) for the diagnosis of at least moderate steatosis (MRI‐PDFF >14.1%). For severe steatosis (MRI‐PDFF>28%) the AUC was 0.92 (95%CI 0.84‐0.99).

### 3.2 Model evaluation

**FIGURE 4:**
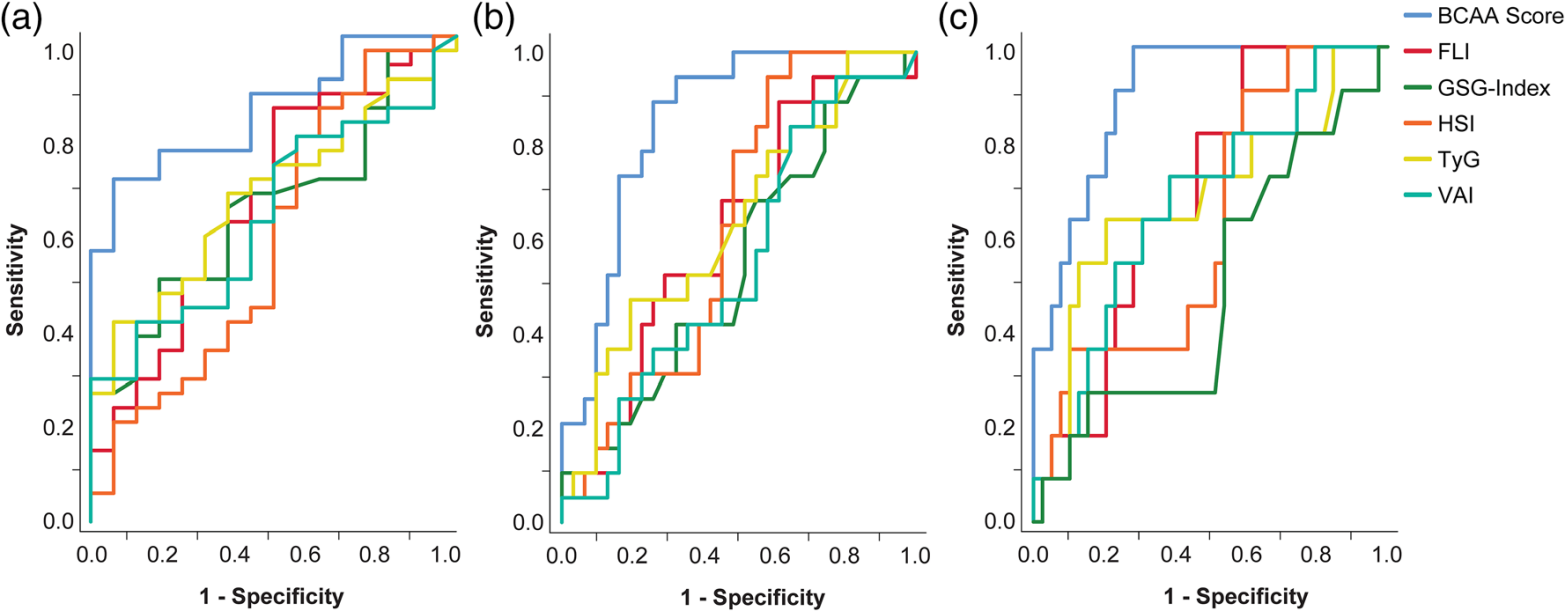
ROC curves evaluating performance of published steatosis scores and the BCAA‐based metabolic score. ROC curves were calculated to determine accuracy of detecting (A) mild steatosis, (B) moderate steatosis and (C) severe steatosis. The BCAA‐based metabolic score showed the highest accuracy in predicting mild, moderate and severe steatosis, respectively. *n* = 52 (68 subjects were analyzed, 16 were excluded due to missing data)

In the next step, we assessed how our model performed compared to previously published scores with c‐statistic. Notably, our model had a higher accuracy than previously proposed scores for the detection of steatosis in children with severe obesity (Figure 4).

The ability of our model to detect patients with at least mild steatosis (>5.1% MRI‐PDFF) was 91.7% (=sensitivity), while specificity to detect patients without the disease was 35%.

Among those who had positive tests for mild steatosis (predicted value >5.1%), the probability of having steatosis (=PPV) was 73.3%. Among those who had negative Test (<5.1%), the probability of being disease free (=NPV) was 75%.

For the detection of at least moderate steatosis, the sensitivity was 71.4%, specificity 85.7%, PPV 75% and NPV 83.3%.

We validated our model in an independent validation cohort of 32 patients (Table S2). Performance of the BCAA‐based model was evaluated by calculating the AUC (Table S3). AUC was 0.82 (95%CI 0.67‐0.97) for the diagnosis of any steatosis (MRI‐PDFF >5.1%) and 0.92 (95%CI 0.83‐1.00) for the diagnosis of at least moderate steatosis (MRI‐PDFF >14.1%). For severe steatosis (MRI‐PDFF>28%) the AUC was 0.90 (95%CI 0.75‐1.00). AUCs were highest for the BCAA‐based model, thus outperforming previously published scores and indices also in the validation cohort and confirming the results of the original cohort.

## 4 DISCUSSION

Here, we show that an incomplex model is able to accurately predict fatty liver. Since childhood obesity is a major health threat worldwide, as is concomitantly NAFLD, our score may be valuable to recognize high‐risk individuals early on to start targeted prevention and treatment strategies. Starting interventions as early as possible is crucial to prevent further progress of liver disease and the development of other comorbidities like type 2 diabetes and cardiovascular disease. Since steatosis is an early predictor of cardiometabolic disease^4^ but can be reliably determined only by biopsy or sophisticated imaging techniques, a simple tool for early recognition and risk stratification as well as for subsequent monitoring as presented here may be of high clinical value. Considering the impact of these disorders for children and adolescents, this notion applies particularly for paediatric patients. Amino acids can readily be quantified from plasma or from dried blood spot specimen, which enable easy sampling and shipping, by metabolic labs. This may open interesting options in particular for disease monitoring and to increase patients' compliance as compared to time‐consuming MRI.

Strikingly, the BCAA‐based metabolic score markedly outperformed proposed indices like HIS, FLI, VAI and TyG for prediction of liver fat content. Besides the inclusion of BCAAs, the better performance of the score presented here can be attributed to the fact that adult NAFLD is markedly different from paediatric NAFLD. ELF test, a relatively cost‐intensive test that combines four different biochemical markers, was also tested as a marker for NAFLD in children with obesity.^40^ However, ELF test performed poorly in our study since it showed no association with steatosis grade and an AUC value of 0.57 (not shown).

The clinical relevance of our score was evaluated with the PPV and NPV. Based on the high PPV and NPV for the detection of mild and especially moderate steatosis, we conclude that our model can differentiate between individuals who have steatosis and those who do not. The ability of our model to detect patients with the disease was 91.7%.

In agreement with our study, previous studies concluded that BCAA dysmetabolism characterizes pathogenesis of NAFLD^18^ and showed that biopsy‐proven liver damage displayed increased values of BCAAs.^14^ Indeed, in our prediction model BCAAs were one of the key factors for predicting hepatic fat accumulation. Thus, our results further support the potential diagnostic value of BCAAs in NAFLD.

Our data do not support a link of inflammation (as determined by TNFα, IL‐6, procalcitonin and CRP values) to BCAA concentrations, thus the mechanisms underlying increased BCAA levels in obesity remain unclear. Decreased expression of catabolic enzymes like branched chain keto acid dehydrogenase kinase may be a factor explaining chronically increased circulating BCAA levels.^30^, ^47^ As a consequence of enhanced BCAA levels, chronic activation of mTOR may be induced leading to increased oxidative stress (ROS) and suppressed autophagy^48^, ^49^ and thus may promote lipid accumulation and lipotoxic liver injury.^27^, ^48^ Therefore, the underlying pathways linking BCAA metabolism to metabolic disease could potentially be pharmacologically targeted to improve hepatic and overall insulin resistance as well as reduction of liver fat content, underlining the utmost importance of further investigations in this field.^30^

## 5 LIMITATIONS

One limitation of the current study is the relatively small sample size. Hence, external validation of our proposed score in bigger cohorts is needed. Since we only included youth with severe obesity from our tertiary care centre in Vienna, Austria, the utility of our score in children with normal weight and of non‐Caucasian ethnicity remains to be determined.

Strengths of this study include its prospective character providing a close correlation between blood sampling and liver MRI, which were all performed within a short time frame (8 weeks). Additionally, strict criteria of inclusion were followed: All patients were extensively tested and excluded from this study if autoimmune, infectious or drug‐induced liver disorder was suspected providing a well‐characterized homogenous cohort. Importantly, all tests were corrected for age, gender and pubertal stage.

## 6 CONCLUSIONS

Elevated circulating BCAAs could be an important link between obesity, NAFLD and other metabolic pathways involved in lipid and glucose metabolism. They may distinguish metabolically healthy from metabolically impaired youth with obesity. A simple BCAA‐based metabolic score predicted steatosis grade in the high‐risk group of children and adolescents with severe obesity and may provide a feasible complement or alternative to current diagnostic measures.

## CONFLICTS OF INTEREST

The authors declare no conflict of interest.

## Supporting information


**TABLE S1** Anthropometric and clinical characteristics of study subjects and correlation with MRI‐PDFF
**TABLE S2** Anthropometric and clinical characteristics of study subjects in the validation cohort (*n* = 32)
**TABLE S3** Performance of the BCAA‐based model in the validation cohort for mild, moderate and severe steatosis

## References

[1] Cohen JC , Horton JD , Hobbs HH . Human fatty liver disease: old questions and new insights. Science (New York, NY). 2011;332:1519‐1523. 10.1126/science.1204265 PMC3229276 21700865

[2] Dunn W , Xu R , Wingard DL , et al. Suspected nonalcoholic fatty liver disease and mortality risk in a population‐based cohort study. Am J Gastroenterol. 2008;103:2263‐2271. 18684196 10.1111/j.1572-0241.2008.02034.x PMC2574666

[3] D'Adamo E , Cali AM , Weiss R , et al. Central role of fatty liver in the pathogenesis of insulin resistance in obese adolescents. Diabetes Care. 2010;33:1817‐1822. 20668154 10.2337/dc10-0284 PMC2909068

[4] Yang S , Kwak S , Lee JH , Kang S , Lee SP . Nonalcoholic fatty liver disease is an early predictor of metabolic diseases in a metabolically healthy population. PLoS One. 2019;14:e0224626. 31682638 10.1371/journal.pone.0224626 PMC6827890

[5] Mangge H , Zelzer S , Pruller F , et al. Branched‐chain amino acids are associated with cardiometabolic risk profiles found already in lean, overweight and obese young. J Nutr Biochem. 2016;32:123‐127. 27142745 10.1016/j.jnutbio.2016.02.007

[6] Corey KE , Kaplan LM . Obesity and liver disease: the epidemic of the twenty‐first century. Clin Liver Dis. 2014;18:1‐18. 24274861 10.1016/j.cld.2013.09.019

[7] Alterio A , Alisi A , Liccardo D , Nobili V . Non‐alcoholic fatty liver and metabolic syndrome in children: a vicious circle. Horm Res Paediatr. 2014;82:283‐289. 25324136 10.1159/000365192

[8] Fedchuk L , Nascimbeni F , Pais R , et al. Performance and limitations of steatosis biomarkers in patients with nonalcoholic fatty liver disease. Aliment Pharmacol Ther. 2014;40:1209‐1222. 25267215 10.1111/apt.12963

[9] Nobili V , Alkhouri N , Alisi A , et al. Nonalcoholic fatty liver disease: a challenge for pediatricians. JAMA Pediatr. 2015;169:170‐176. 25506780 10.1001/jamapediatrics.2014.2702

[10] Koplan JP , Liverman CT , Kraak VI . Preventing childhood obesity: health in the balance. In: Youth IoMCoPoOiCa , ed. The National Academies Collection: reports funded by National Institutes of Health. Washington, DC: National Academy of Sciences; 2005. 22379642

[11] Schwimmer JB , Middleton MS , Behling C , et al. Magnetic resonance imaging and liver histology as biomarkers of hepatic steatosis in children with nonalcoholic fatty liver disease. Hepatology (Baltimore, MD). 2015;61:1887‐1895. 10.1002/hep.27666 PMC4670559 25529941

[12] Bi WR , Yang CQ , Shi Q , et al. Large‐scale analysis of factors influencing nonalcoholic fatty liver disease and its relationship with liver enzymes. Genet Mol Res. 2014;13:5880‐5891. 25117346 10.4238/2014.August.7.3

[13] Wong VW , Wong GL , Tsang SW , et al. Metabolic and histological features of non‐alcoholic fatty liver disease patients with different serum alanine aminotransferase levels. Aliment Pharmacol Ther. 2009;29:387‐396. 19035982 10.1111/j.1365-2036.2008.03896.x

[14] Gaggini M , Carli F , Rosso C , et al. Altered amino acid concentrations in NAFLD: impact of obesity and insulin resistance. Hepatology (Baltimore, MD). 2018;67:145‐158. 10.1002/hep.29465 28802074

[15] Haufe S , Witt H , Engeli S , et al. Branched‐chain and aromatic amino acids, insulin resistance and liver specific ectopic fat storage in overweight to obese subjects. Nutr Metab Cardiovasc Dis. 2016;26:637‐642. 27134061 10.1016/j.numecd.2016.03.013

[16] Kaikkonen JE , Wurtz P , Suomela E , et al. Metabolic profiling of fatty liver in young and middle‐aged adults: cross‐sectional and prospective analyses of the Young Finns Study. Hepatology (Baltimore, MD). 2017;65:491‐500. 10.1002/hep.28899 PMC5299457 27775848

[17] Cheng S , Wiklund P , Autio R , et al. Adipose tissue dysfunction and altered systemic amino acid metabolism are associated with non‐alcoholic fatty liver disease. PLoS One. 2015;10:e0138889. 26439744 10.1371/journal.pone.0138889 PMC4595021

[18] Goffredo M , Santoro N , Trico D , et al. A branched‐chain amino acid‐related metabolic signature characterizes obese adolescents with non‐alcoholic fatty liver disease. Nutrients. 2017;9:642. 10.3390/nu9070642 PMC5537762 28640216

[19] Koch M , Freitag‐Wolf S , Schlesinger S , et al. Serum metabolomic profiling highlights pathways associated with liver fat content in a general population sample. Eur J Clin Nutr. 2017;71:995‐1001. 28378853 10.1038/ejcn.2017.43

[20] Newgard CB . Metabolomics and metabolic diseases: where do we stand? Cell Metab. 2017;25:43‐56. 28094011 10.1016/j.cmet.2016.09.018 PMC5245686

[21] Newgard CB , An J , Bain JR , et al. A branched‐chain amino acid‐related metabolic signature that differentiates obese and lean humans and contributes to insulin resistance. Cell Metab. 2009;9:311‐326. 19356713 10.1016/j.cmet.2009.02.002 PMC3640280

[22] Wang TJ , Larson MG , Vasan RS , et al. Metabolite profiles and the risk of developing diabetes. Nat Med. 2011;17:448‐453. 21423183 10.1038/nm.2307 PMC3126616

[23] Yang P , Hu W , Fu Z , et al. The positive association of branched‐chain amino acids and metabolic dyslipidemia in Chinese Han population. Lipids Health Dis. 2016;15:120. 27457614 10.1186/s12944-016-0291-7 PMC4960685

[24] Ruiz‐Canela M , Toledo E , Clish CB , et al. Plasma branched‐chain amino acids and incident cardiovascular disease in the PREDIMED Trial. Clin Chem. 2016;62:582‐592. 26888892 10.1373/clinchem.2015.251710 PMC4896732

[25] Shah SH , Bain JR , Muehlbauer MJ , et al. Association of a peripheral blood metabolic profile with coronary artery disease and risk of subsequent cardiovascular events. Circ Cardiovasc Genet. 2010;3:207‐214. 20173117 10.1161/CIRCGENETICS.109.852814

[26] McCormack SE , Shaham O , McCarthy MA , et al. Circulating branched‐chain amino acid concentrations are associated with obesity and future insulin resistance in children and adolescents. Pediatr Obes. 2013;8:52‐61. 22961720 10.1111/j.2047-6310.2012.00087.x PMC3519972

[27] Zhang F , Zhao S , Yan W , et al. Branched chain amino acids cause liver injury in obese/diabetic mice by promoting adipocyte lipolysis and inhibiting hepatic autophagy. EBioMedicine. 2016;13:157‐167. 27843095 10.1016/j.ebiom.2016.10.013 PMC5264279

[28] Breitman I , Saraf N , Kakade M , et al. The effects of an amino acid supplement on glucose homeostasis, inflammatory markers, and incretins after laparoscopic gastric bypass. J Am Coll Surg. 2011;212:617‐625. discussion 625‐617. 21463799 10.1016/j.jamcollsurg.2010.12.040 PMC3230243

[29] Mu WC , VanHoosier E , Elks CM , Grant RW . Long‐term effects of dietary protein and branched‐chain amino acids on metabolism and inflammation in mice. Nutrients. 2018;10:918. 10.3390/nu10070918 PMC6073443 30021962

[30] White PJ , McGarrah RW , Grimsrud PA , et al. The BCKDH kinase and phosphatase integrate BCAA and lipid metabolism via regulation of ATP‐citrate lyase. Cell Metab. 2018;27:1281‐1293.e1287. 29779826 10.1016/j.cmet.2018.04.015 PMC5990471

[31] Nobili V , Alisi A , Newton KP , Schwimmer JB . Comparison of the phenotype and approach to pediatric vs adult patients with nonalcoholic fatty liver disease. Gastroenterology. 2016;150:1798‐1810. 27003600 10.1053/j.gastro.2016.03.009 PMC4887388

[32] Kalia HS , Gaglio PJ . The prevalence and pathobiology of nonalcoholic fatty liver disease in patients of different races or ethnicities. Clin Liver Dis. 2016;20:215‐224. 27063265 10.1016/j.cld.2015.10.005

[33] Kalarchian MA , Levine MD , Arslanian SA , et al. Family‐based treatment of severe pediatric obesity: randomized, controlled trial. Pediatrics. 2009;124:1060‐1068. 19786444 10.1542/peds.2008-3727 PMC2935494

[34] Kalarchian MA , Levine MD , Marcus MD . Structured dietary interventions in the treatment of severe pediatric obesity: a pilot study. Bariatr Surg Pract Patient Care. 2013;8:58‐60. 24761366 10.1089/bari.2013.9990 PMC3827847

[35] Kromeyer‐Hauschild K , Wabitsch M , Kunze D , et al. Perzentile für den Body‐mass‐Index für das Kindes‐ und Jugendalter unter Heranziehung verschiedener deutscher Stichproben. Monatsschr Kinderheilkd. 2001;149:807‐818.

[36] Matthews DR , Hosker JP , Rudenski AS , Naylor BA , Treacher DF , Turner RC . Homeostasis model assessment: insulin resistance and beta‐cell function from fasting plasma glucose and insulin concentrations in man. Diabetologia. 1985;28:412‐419. 3899825 10.1007/BF00280883

[37] Sirlin CB . Invited commentary. RadioGraphics. 2009;29:1277‐1280. 19764109 10.1148/027153330290051277

[38] Pickhardt PJ , Graffy PM , Reeder SB , Hernando D , Li K . Quantification of liver fat content with unenhanced MDCT: phantom and clinical correlation with MRI proton density fat fraction. AJR Am J Roentgenol. 2018;211:W151‐w157. 30016142 10.2214/AJR.17.19391 PMC6615548

[39] Kühn JP , Meffert P , Heske C , et al. Prevalence of fatty liver disease and hepatic Iron overload in a northeastern German population by using quantitative MR imaging. Radiology. 2017;284:706‐716. 28481195 10.1148/radiol.2017161228 PMC5565690

[40] Szybowska P , Wojcik M , Starzyk JB , Sztefko K . Enhanced liver fibrosis (ELF) test in obese children with ultrasound‐proven liver steatosis. Neuro Endocrinol Lett. 2015;36:700‐705. 26859594

[41] Bedogni G , Bellentani S , Miglioli L , et al. The fatty liver index: a simple and accurate predictor of hepatic steatosis in the general population. BMC Gastroenterol. 2006;6:33. 17081293 10.1186/1471-230X-6-33 PMC1636651

[42] Lee JH , Kim D , Kim HJ , et al. Hepatic steatosis index: a simple screening tool reflecting nonalcoholic fatty liver disease. Dig Liver Dis. 2010;42:503‐508. 19766548 10.1016/j.dld.2009.08.002

[43] Amato MC , Giordano C , Galia M , et al. Visceral adiposity index: a reliable indicator of visceral fat function associated with cardiometabolic risk. Diabetes Care. 2010;33:920‐922. 20067971 10.2337/dc09-1825 PMC2845052

[44] Zhang S , Du T , Zhang J , et al. The triglyceride and glucose index (TyG) is an effective biomarker to identify nonalcoholic fatty liver disease. Lipids Health Dis. 2017;16:15. 28103934 10.1186/s12944-017-0409-6 PMC5248473

[45] Mehmood T , Liland KH , Snipen L , Sæbø S . A review of variable selection methods in partial least squares regression. Chemom Intell Lab Syst. 2012;118:62‐69.

[46] Akarachantachote N , Chadcham S , Saithanu K . Cutoff threshold of variable importance in projection for variable selection. Int J Pure Appl Math. 2014;94:307‐322.

[47] Herman MA , She P , Peroni OD , Lynch CJ , Kahn BB . Adipose tissue branched chain amino acid (BCAA) metabolism modulates circulating BCAA levels. J Biol Chem. 2010;285:11348‐11356. 20093359 10.1074/jbc.M109.075184 PMC2857013

[48] Liu TY , Xiong XQ , Ren XS , et al. FNDC5 alleviates hepatosteatosis by restoring AMPK/mTOR‐mediated autophagy, fatty acid oxidation, and lipogenesis in mice. Diabetes. 2016;65:3262‐3275. 27504012 10.2337/db16-0356

[49] Sanguesa G , Roglans N , Baena M , et al. mTOR is a key protein involved in the metabolic effects of simple sugars. Int J Mol Sci. 2019;20.1117. 10.3390/ijms20051117 PMC6429387 30841536

