# Supplementary material for: A branched‐chain amino acid‐based metabolic score can predict liver fat in children and adolescents with severe obesity
Source: Pediatr Obes. 2020 Oct 14;16(4):e12739. doi: 10.1111/ijpo.12739 (PMC7988615; doi:10.1111/ijpo.12739)
Supplement: Supplementary file 1 — TABLE S1 Anthropometric and clinical characteristics of study subjects and correlation with MRI‐PDFF TABLE S2 Anthropometric and clinical characteristics of study subjects in the validation cohort (n = 32) TABLE S3 Performance of the BCAA‐based model in the validation cohort for mild, moderate and severe steatosis [file IJPO-16-e12739-s001.docx]

**Supplementary Material**

Supplementary Table 1. Anthropometric and clinical characteristics of study subjects and correlation with MRI-PDFF

|  |  |  |  |  |
| --- | --- | --- | --- | --- |
|  |  |  | **Correlation with MRI-PDFF (R=)** | **p-value** |
| Age |  |  | 0.01 | n.s. |
| BMI z-score |  |  | 0.06 | n.s. |
| Waist circumference (cm) | |  | 0.2 | n.s. |
| Hip circumference (cm) | |  | 0.15 | n.s. |
| BIA (body fat%) |  |  | 0.03 | n.s. |
|  |  |  |  |  |
| HOMA-IR |  |  | 0.53 | <0.01 |
| HOMA-β |  |  | 0.58 | <0.01 |
| Fasting glucose (mg/dl) | |  | 0.02 | n.s. |
| Insulin (µU/ml) | |  | 0.56 | <0.01 |
| Ferritin (µg/l) |  |  | 0.36 | <0.01 |
| Platlet count (10^9/L) | |  | 0.07 | n.s. |
| Alkaline phosphatase (U/l) | |  | 0.11 | n.s. |
| GGT (U/l) |  |  | 0.43 | <0.01 |
| ALT (U/l) |  |  | 0.62 | <0.01 |
| AST (U/l) |  |  | 0.5 | <0.01 |
| Triglycerides (mg/dl) | |  | 0.31 | 0.02 |
| Total cholesterol (mg/dl) | |  | 0.28 | 0.02 |
| HDL-C (mg/dl) | |  | 0.2 | n.s. |
| Uric acid (mg/dl) | |  | 0.21 | n.s. |
| ELF (Enhanced liver fibrosis test) | |  | <0.01 | n.s. |
|  |  |  |  |  |
| CRP (mg/dl) |  |  | -0.01 | n.s. |
| IL-6 (pg/ml) |  |  | 0.02 | n.s. |
| Procalcitonin (ng/ml) | |  | 0.29 | 0.02 |
| TNFα (pg/ml) | |  | 0.33 | 0.02 |
| CK-18 (U/ll) | |  | 0.45 | <0.01 |
| BCAA (µmol/l) | |  | 0.46 | <0.01 |
| Correlations are Pearson's and Spearman's coefficient for normal distributed and skewed variables, respectively. BMI, body mass index; BIA, bioelectrical impedance analysis; HOMA-IR, Homeostatic Model Assessment of Insulin Resistance; HOMA-β, Homeostatic Model Assessment of liver insulin resistance | | | | |

**Supplementary Table 2. Anthropometric and clinical characteristics of study subjects in the validation cohort (n=32)**

|  |  | **No steatosis  (n=15)** | **Mild steatosis  (n=7)** | **Moderate steatosis (n=5)** | **Severe steatosis (n=5)** | **p-value** |
| --- | --- | --- | --- | --- | --- | --- |
| Gender | female/male | 7/8 | 4/3 | 0/5 | 1/4 | n.s. |
|  |  |  |  |  |  |  |
| Age |  | 13.00 (3.00) | 14.00 (3.00) | 12.00 (2.00) | 12.00 (2.00) | n.s. |
| BMI z-score |  | 2.75 (0.56) | 2.91 (0.40) | 3.04 (0.45) | 2.74 (0.16) | n.s. |
| Waist circumference (cm) | | 103.70 (12.10 ) | 104.50 (15.60) | 118.30 (20.30) | 101.80 (13.30) | n.s. |
| Hip circumference (cm) | | 109.60 (12.70 ) | 112.90 (14.80) | 116.30 (12.80) | 105.20 (12.90) | n.s. |
| BIA (body fat%) | | 43.26 (7.40 ) | 45.35 (7.23) | 47.30 (11.06) | 40.10 (11.84) | n.s. |
|  | |  |  |  |  |  |
| HOMA-IR |  | 4.68 (2.57, 6.83) | 5.93 (4.15, 10.33) | 7.54 (6.26, 12.72) | 15.59 (13.02, 21.45) | 0.02 |
| HOMA-β |  | 4.98 (2.55, 8.81) | 8.72 (4.52, 9.91) | 12.38 (7.87, 12.94) | 12.82 (3.63, 19.57) | n.s. |
| Fasting glucose (mg/dl) | | 87.00 (5.00 ) | 87.00 (8.00) | 81.00 (7.00) | 100.00 (11.00) | <0.01 |
| Insulin (µU/ml) | | 25.45 (15.49) | 30.13 (13.40) | 47.62 (28.65) | 69.28 (39.37) | <0.01 |
| Ferritin (µg/l) |  | 48.75 (23.33) | 65.97 (41.20) | 104.60 (65.47) | 106.08 (70.07) | n.s. |
| Platlet count (10^9/L) | | 322.00 (60.00) | 303.81 (66.00) | 310.00 (121.00) | 375.00 (117.00) | n.s. |
| Alkaline phosphatase (U/l) | | 156.20 (56.65) | 162.00 (54.00) | 147.00 (57.00) | 214.00 (61.00) | n.s. |
| GGT (U/l) |  | 26.00 (7.00) | 32.00 (12.00) | 29.00 (12.14) | 40.40 (28.01) | <0.01 |
| ALT (U/l) |  | 21.07 (5.87) | 39.00 (30.03) | 47.00 (21.83) | 74.60 (43.78) | <0.01 |
| AST (U/l) |  | 17.93 (4.99) | 23.00 (8.04) | 34.00 (6.00) | 48.00 (24.00) | 0.02 |
| Triglycerides (mg/dl) | | 94.00 (71.00, 110.00) | 133.00 (119.00, 190.00) | 84.00 (79.00, 96.00) | 112.00 (104.00, 380.00) | n.s. |
| Total cholesterol (mg/dl) | | 156.00 (136.00, 187.00) | 184.00 (167.00, 191.00) | 140.00 (129.00, 146.00) | 172.00 (164.00, 197.00) | n.s. |
| HDL-C (mg/dl) | | 46.00 (38.00, 55.00) | 43.00 (36.00, 61.00) | 40.00 (39.00, 43.00) | 36.00 (31.00, 37.00) | n.s. |
| Uric acid (mg/dl) | | 5.10 (1.08 ) | 5.57 (1.38) | 7.30 (0.94) | 6.64 (1.75) | n.s. |
| ELF test | | 8.32( 0.55 ) | 8.60 (0.46) | 8.91 (0.86) | 8.70 (0.38) | n.s. |
|  | |  |  |  |  |  |
| CRP (mg/dl) |  | 0.45 (0.27, 1.25) | 0.41 (0.24, 0.91) | 0.80 (0.53, 1.15) | 0.18 (0.17, 0.57) | n.s. |
| IL-6 (pg/ml) |  | 3.91 (2.70, 5.20) | 3.76 (2.61, 4.96) | 5.34 (4.53, 7.01) | 3.63 (2.64, 4.73) | n.s. |
| Procalcitonin (ng/ml) | | 0.01 (0.01, 0.02) | 0.04 (0.03, 0.05) | 0.03 (0.03, 0.04) | 0.06 (0.05, 0.09) | 0.02 |
| TNFα (pg/ml) | | 0.75 (0.65, 0.95) | 0.98 (0.78, 1.10) | 1.08 (0.85, 1.30) | 1.30 (1.10, 1.50) | n.s. |
| CK-18 (U/l) | | 97.53 (91.60, 120.59) | 136.82 (132.04, 157.42) | 256.53 (242.16, 270.90) | 129.92 (114.98, 234.54) | <0.01 |
| BCAA (µmol/l) | | 482.46 (83.29) | 503.86 (48.25) | 501.76 (59.89) | 518.21 (90.35) | n.s. |
|  | | | | | | |
| Values are means and (standard deviation) for normally distributed variables and median (25^th^, 75^th^ percentile) for skewed variables. P-values less than 0.05 was considered significant and were determined by ANOVA or Kruskall Wallis test, respectively. BMI, body mass index; BIA (body fat%), body fat in % determined by bioelectrical impedance analysis; HOMA-IR, Homeostatic Model Assessment of Insulin Resistance; HOMA-β, Homeostatic Model Assessment of liver insulin resistance, AP: Alkaline phosphatase, ELF: Enhanced liver fibrosis | | | | | | |

**Supplementary Table 3. Performance of the BCAA-based model in the validation cohort for mild, moderate and severe steatosis**

| **Mild steatosis (MRI-PDFF >5.1%)** | | | |
| --- | --- | --- | --- |
| **Indices** | **AUC** | **95% CI** | |
| BCAA-based metabolic score | 0.82 | 0.67-0.97 | |
| FLI | 0.69 | 0.50-0.89 | |
| GSG-Index | 0.71 | 0.54-0.89 | |
| HSI | 0.74 | 0.56-0.93 | |
| TyG | 0.72 | 0.54-0.91 | |
| VAI | 0.61 | 0.41-0.81 | |
|  |  |  |  |
| **Moderate steatosis (MRI-PDFF >14.1%)** | | | |
| **Indices** | **AUC** | **95% CI** | |
| BCAA-based metabolic score | 0.92 | 0.83-1.00 | |
| FLI | 0.66 | 0.47-0.86 | |
| GSG-Index | 0.82 | 0.67-0.97 | |
| HSI | 0.71 | 0.52-0.89 | |
| TyG | 0.53 | 0.30-0.76 | |
| VAI | 0.52 | 0.30-0.75 | |
|  |  |  |  |
| **Severe steatosis (MRI-PDFF >28%)** | | | |
| **Indices** | **AUC** | **95% CI** | |
| BCAA-based metabolic score | 0.90 | 0.75-1.00 | |
| FLI | 0.58 | 0.33-0.83 | |
| GSG-Index | 0.80 | 0.59-1.00 | |
| HSI | 0.59 | 0.36-0.81 | |
| TyG | 0.76 | 0.53-1.00 | |
| VAI | 0.73 | 0.45-1.00 | |
